# Assessment of Humoral Immune Responses to Blood-Stage Malaria Antigens following ChAd63-MVA Immunization, Controlled Human Malaria Infection and Natural Exposure

**DOI:** 10.1371/journal.pone.0107903

**Published:** 2014-09-25

**Authors:** Sumi Biswas, Prateek Choudhary, Sean C. Elias, Kazutoyo Miura, Kathryn H. Milne, Simone C. de Cassan, Katharine A. Collins, Fenella D. Halstead, Carly M. Bliss, Katie J. Ewer, Faith H. Osier, Susanne H. Hodgson, Christopher J. A. Duncan, Geraldine A. O’Hara, Carole A. Long, Adrian V. S. Hill, Simon J. Draper

**Affiliations:** 1 The Jenner Institute, University of Oxford, Oxford, United Kingdom; 2 Laboratory of Malaria and Vector Research, NIAID/NIH, Rockville, Maryland, United States of America; 3 KEMRI Centre for Geographic Medicine Research, Kilifi, Kenya; 4 Centre for Clinical Vaccinology and Tropical Medicine, The Jenner Institute, University of Oxford, Churchill Hospital, Oxford, United Kingdom; Queensland Institute of Medical Research, Australia

## Abstract

The development of protective vaccines against many difficult infectious pathogens will necessitate the induction of effective antibody responses. Here we assess humoral immune responses against two antigens from the blood-stage merozoite of the *Plasmodium falciparum* human malaria parasite – MSP1 and AMA1. These antigens were delivered to healthy malaria-naïve adult volunteers in Phase Ia clinical trials using recombinant replication-deficient viral vectors – ChAd63 to prime the immune response and MVA to boost. In subsequent Phase IIa clinical trials, immunized volunteers underwent controlled human malaria infection (CHMI) with *P. falciparum* to assess vaccine efficacy, whereby all but one volunteer developed low-density blood-stage parasitemia. Here we assess serum antibody responses against both the MSP1 and AMA1 antigens following i) ChAd63-MVA immunization, ii) immunization and CHMI, and iii) primary malaria exposure in the context of CHMI in unimmunized control volunteers. Responses were also assessed in a cohort of naturally-immune Kenyan adults to provide comparison with those induced by a lifetime of natural malaria exposure. Serum antibody responses against MSP1 and AMA1 were characterized in terms of i) total IgG responses before and after CHMI, ii) responses to allelic variants of MSP1 and AMA1, iii) functional growth inhibitory activity (GIA), iv) IgG avidity, and v) isotype responses (IgG1-4, IgA and IgM). These data provide the first in-depth assessment of the quality of adenovirus-MVA vaccine-induced antibody responses in humans, along with assessment of how these responses are modulated by subsequent low-density parasite exposure. Notable differences were observed in qualitative aspects of the human antibody responses against these malaria antigens depending on the means of their induction and/or exposure of the host to the malaria parasite. Given the continued clinical development of viral vectored vaccines for malaria and a range of other diseases targets, these data should help to guide further immuno-monitoring studies of vaccine-induced human antibody responses.

## Introduction

*Plasmodium falciparum* is the preeminent cause of human malaria disease and a leading example of a parasite with a complex multi-host multi-stage lifecycle where a number of steps in the infectious process have been shown to be antibody-susceptible. These include the invasion of liver cells by sporozoites delivered from the mosquito bite; the invasion of red blood cells (RBC) by merozoites; clearance of infected RBC; and sexual-stage development within the blood meal inside the mosquito midgut [1]. However, despite tremendous efforts, the development of a highly effective subunit vaccine against infection, disease or transmission has proved an elusive goal, and *P. falciparum* continues to exert a huge burden on global public health in terms of morbidity and mortality [2], as well as financially in terms of maintaining effective control and intervention measures [3]. Such difficulties, with regard to subunit vaccine development have arisen through a variety of reasons, including factors relating to the complex biology of the parasite’s lifecycle coupled with an incomplete understanding of protective immune effector mechanisms that function *in vivo* in humans [4].

One leading strategy for many years has sought to develop an effective antibody-inducing vaccine against the blood-stage merozoite, seeking to neutralize RBC invasion [5]. For a number of decades, merozoite surface protein 1 (MSP1) [6] and apical membrane antigen 1 (AMA1) [7] have been assessed as leading subunit vaccine candidate antigens – both are expressed by the invasive blood-stage merozoite, with evidence they are also present at the late liver-stage [8] or sporozoite stage [9] of the parasite lifecycle. These antigens, most often delivered as antibody-inducing recombinant protein formulated in adjuvant, have shown disappointing levels of efficacy in Phase IIa/b clinical trials [5], [10], [11], although one AMA1 formulation was reported to show strain-specific efficacy in a Phase IIb field trial in Malian children [12]. Consequently, a number of researchers within the field have argued that an effective blood-stage vaccine would in fact necessitate the induction of T cell responses, in conjunction with antibody responses, in order to achieve protection [13]. In more recent years, recombinant simian adenoviral and poxviral vectored vaccines have been developed as a highly immunogenic delivery platform with a favourable safety profile for human use [14]–[16]. In particular the use of recombinant adenovirus vectors to prime immune responses, followed by immunization with a recombinant poxvirus to boost immune responses, has emerged as a leading strategy across multiple fields including various infectious diseases and cancer. Although traditionally regarded as a platform for inducing strong cellular immunity, this specific vectored vaccine approach has also proved highly suited for antibody induction, so long as the transgenes are designed and the vectors are deployed in a suitable manner [17], [18].

Recently we assessed this approach for targeting the blood-stage malaria antigens *P. falciparum* MSP1 and AMA1. In a series of Phase Ia clinical trials, we demonstrated that the use of a recombinant chimpanzee adenovirus serotype 63 (ChAd63) prime, followed by a boost with the recombinant orthopoxvirus, modified vaccinia virus Ankara (MVA), was safe in healthy UK adults and capable of inducing strong cellular and humoral immune responses against both antigens [19], [20]. However, in a subsequent series of Phase IIa clinical trials, where immunized volunteers were exposed to five infectious mosquito bites, almost no vaccine efficacy was observed with only one volunteer protected who had been co-immunized with the vectors encoding MSP1 and AMA1 [21]. Nevertheless, the use of the controlled human malaria infection (CHMI) model in these studies did allow for the impact of blood-stage parasite exposure to be assessed on vaccine-induced immune responses in non-protected volunteers. Moreover, the adenovirus-MVA regimen remains a leading platform in clinical development for a wide range of disease indications and pathogens, including other stages of the malaria parasite lifecycle [22], [23], human immunodeficiency virus-1 (HIV-1) [24], hepatitis C virus [25], leishmaniasis [26] and cancer amongst others. In a growing number of cases, it is likely that antibodies, in conjunction with cellular immunity, may contribute to effective immunity. We have previously reported in detail the cellular immune responses (T cell and B cell) induced by these vaccines, and the impact of CHMI on these responses [27], [28]. Here we now sought to characterize humoral antibody immune responses in humans in much greater detail following ChAd63-MVA immunization with two different antigens including assessment of serum antibody avidity and isotype profiles. We also explored the impact of CHMI on vaccine-induced antibody titers, avidity and isotype profiles and provide comparison to *de novo* antigen-specific responses induced in control volunteers as opposed to those induced by a lifetime of exposure in naturally-immune Kenyan adults. These data provide the first in-depth assessment of the quality of adenovirus-MVA vaccine-induced antibody responses in humans, along with assessment of how these responses can be modulated by subsequent parasite exposure.

## Materials and Methods

### Immunization Groups and Serum Samples

Serum samples used throughout this study were obtained from Phase Ia safety and immunogenicity clinical trials for the MSP1 [19] and AMA1 [20] candidate vaccines, or from Phase IIa efficacy studies where immunized and non-immunized infectivity control volunteers underwent CHMI with vaccine homologous *P. falciparum* 3D7 clone sporozoites delivered by five infectious mosquito bites. In the case of the Phase IIa studies, vaccinated volunteers were immunized with vaccines encoding MSP1, AMA1 or the liver-stage malaria antigen ME-TRAP (a multi-epitope string fused to the thrombospondin-related adhesion protein) [29] either administered alone, or co-administered together at separate sites (MSP1+AMA1 or MSP1+ME-TRAP) [21], [22]. Outlines of the immunization and bleed schedules for each type of trial are shown in Figure S1. In all cases, the antigens were separately delivered by a heterologous prime-boost immunization regimen consisting of a priming intramuscular (i.m.) vaccination with a recombinant replication-deficient ChAd63 vector (doses ranging from 5×10^9^–5×10^10^ viral particles (vp)), followed eight weeks later by a boosting vaccination i.m. with the MVA vector (doses ranging from 1×10^8^–5×10^8^ plaque forming units (pfu)) recombinant for the same antigen, all as previously described [19]–[22].

All necessary ethical approvals (from the UK Gene Therapy Advisory Committee, Oxfordshire Research Ethics Committee or Berkshire Research Ethics Committee) and regulatory approvals (from the UK Medicines and Healthcare products Regulatory Agency, MHRA) were granted as previously described, and the four trials were registered with ClinicalTrials.gov (NCT01095055; NCT01003314; NCT01142765; and NCT00890760) [19]–[22]. All volunteers gave written informed consent prior to participation, and the studies were conducted according to the principles of the Declaration of Helsinki and in accordance with Good Clinical Practice (GCP). All volunteers participating in these clinical trials gave consent for samples to be used for exploratory immunology analysis. Volunteer information was anonymized for all immunological analyses. Serum samples from the trials were all prepared and frozen according to a previously described protocol [19].

Serum from adults living in Kenya was collected during adult cross-sectional surveys between 2006 and 2008 from the villages surrounding the Chonyi area in Kilifi, Kenya which experiences moderate malaria transmission with an entomological inoculation rate (EIR) of 10–100 infective bites/person/year [30]. These adults are considered to have substantial naturally-acquired immunity as evidenced by the decline in clinical episodes of malaria with age [31]. Scientific and ethical approvals for the Kenyan serum samples were granted by the Kenya National Scientific and Research Ethics Committees respectively, SSC No. 1131.

### Vaccine Antigens

The composition of the bi-allelic vaccine inserts for MSP1 [32] and AMA1 [33], [34] used in the ChAd63 and MVA vaccine vectors have been previously described. In the case of AMA1, a bivalent transgene was optimized to consist of the 3D7 and FVO strain alleles fused in tandem; whilst for MSP1 an insert was designed comprising both the 3D7/MAD20 and K1/Wellcome alleles of the dimorphic 42 kDa C-terminal region (MSP1_42_/sequence blocks 16+17) fused in tandem and preceded by the naturally conserved regions of MSP1 sequence (blocks 1, 3, 5 and 12). The MSP1_42_ region is composed of an N-terminal 33 kDa region (MSP1_33_, block 16) followed by a C-terminal 19 kDa region (MSP1_19_, block 17). In the case of ME-TRAP, the construct consists of the T9/96 allele of *P. falciparum* TRAP fused to a multi-epitope (ME) string [35].

### ELISA Antigens

The ELISA antigens used in this study have been previously described [19], [20]. Unless otherwise stated, proteins representing the 3D7 allele of the antigen were used. Briefly, recombinant MSP1_19_ (ETSR = 3D7/MAD20 allele or QNKG = K1/Wellcome allele) was produced in and purified from *Escherichia coli* as a soluble glutathione *S-*transferase (GST) fusion protein [32]. Previous data have shown negative responses against GST control protein in serum analysed in these clinical trials. Recombinant 3D7 AMA1 protein was a kind gift from Dr Chetan Chitnis (ICGEB, New Delhi, India) and FVO AMA1 was a kind gift from Dr Mike Blackman (NIMR, London, UK) [33].

### Total IgG ELISA

Standardized ELISAs were conducted according to previously described protocols for both MSP1_19_ and AMA1 [19], [20], [36]. Briefly, a reference standard human serum sample for each test antigen (with a relatively high antigen-specific response) was serially diluted to generate a standard curve on each ELISA plate. This standard sample was assigned a value in arbitrary ELISA units (AU), defined as the dilution giving an optical density at 405 nm (OD_405_) = 1.0. The standard curve was then used to convert absorbance values of individual test sera (diluted to fall within the linear range of the standard curve) into AU (Gen5 ELISA software v1.10, BioTek, UK). The limit of detection in both ELISA assays was 10 AU, and we assigned the AU value of 1.0 for any test samples with less than 10 AU. Any values more than 10 AU are considered as positive responses in the analyses reported here.

### IgG Antibody Avidity ELISA

IgG antibody avidity was assessed by sodium thiocyanate (NaSCN)-displacement ELISA. The assays were performed exactly as for total IgG except sera were individually diluted in Casein block solution (Pierce, UK) (using previously measured total IgG ELISA AU titers) to a level calculated to give an OD_405_ = 1.0, and plated at 50 µL/well in 16 wells of a 96 well plate. Following incubation and washing, an ascending concentration of the chaotropic agent NaSCN was added in duplicate wells (50 µL per well) down the plate (0 to 7 Molar (M) NaSCN). The NaSCN gradient was prepared beforehand by diluting an 8 M stock solution (made using water) into PBS. Plates were incubated for 15 min at room temperature (RT) before washing and development as for total IgG. The intercept of the OD_405_ curve for each sample with the line of 50% reduction of the OD_405_ in the NaSCN-free well for each sample (i.e. the concentration of NaSCN required to reduce the OD_405_ to 50% of that without NaSCN = IC50) was used as a measure of avidity.

### Antibody Isotype ELISA

Antibody isotype ELISAs were performed exactly as for total IgG except sera were individually diluted 1∶100 in Casein block solution and 50 µL added to duplicate wells of six 96 well plates (one for each isotype analysis). After incubating for 2 h the plates were washed and the following secondary antibodies added (one per plate) at 1∶1000 dilution, 50 µL per well, in Casein block solution: biotin-conjugated mouse anti-human IgG1 Fc (clone HP6070) (Life Technologies, UK); biotin-conjugated mouse anti-human IgG2 Fc (clone HP6002) (Life Technologies, UK); biotin-conjugated mouse anti-human IgG3 (clone HP6050) (Sigma, UK); biotin-conjugated mouse anti-human IgG4 (clone HP6025) (Sigma, UK); alkaline phosphatase-conjugated goat polyclonal anti-human IgA α-chain (Sigma, UK); and biotin-conjugated goat polyclonal anti-human IgM µ-chain (Sigma, UK). After 1 h, all the plates were washed before addition of 50 µL per well ExtrAvidin alkaline phosphatase (Sigma, UK) diluted 1∶5000 in Casein block solution (to all plates except IgA, to which blocking solution only was added). After 30 min, all plates were washed again before development as per the total IgG ELISA. Blank wells and internal development controls were included on each plate.

When coating the plates with recombinant MSP1_19_ or AMA1 antigen, a number of controls (2 wells per control, 50µL per well) were included on each plate. Instead of coating with recombinant malaria protein, these controls wells were coated instead with native human IgG1, IgG2, IgG3 and IgG4 (AbD Serotec, UK) at 2 µg/mL in PBS; or a hyperimmune Kenyan adult serum sample diluted 1∶10000 in PBS. During the ELISA, these wells were filled with Casein block solution during the steps where test sera were added to the rest of the plate. In the case of the MSP1_19_ ELISAs, further controls included the addition of epitope-matched monoclonal antibodies (mAbs) in the place of test sera (again 2 wells per control) at 0.2 µg/mL in Casein block solution. These mAbs were against the e9 epitope of *P. falciparum* MSP1_19_ and possessed the human IgG1 [37], IgG3 (Llewellyn *et al.*, in preparation) or IgA1 [38] Fc regions [a kind gift from Prof Richard Pleass, Liverpool School of Tropical Medicine, Liverpool, UK]. Each of the above control reagents was used to confirm the specificity of the isotype-specific secondary antibodies, and in order for an assay to pass quality control analysis, each control was required to give a positive signal for the relevant secondary antibody, and no response above background to the others (data not shown). During the development of this assay, some secondary antibody reagents did not show sufficient isotype-specific reactivity against these internal controls, and thus were replaced with alternative reagents (data not shown).

### Assay of Growth Inhibitory Activity (GIA)

GIA was assessed as previously described [19], [39] with a standardized assay using purified IgG. Briefly, each test IgG (10 mg/mL in a final test well) was incubated with synchronized *P. falciparum* parasites for 48 h and relative parasitemia levels were quantified by biochemical determination of parasite lactate dehydrogenase.

### Statistical analysis

Data were analysed using GraphPad Prism version 5.04 for Windows (GraphPad Software Inc., California, USA) and non-parametric analyses. To compare responses between selected time-points in a group a Wilcoxon matched-pairs signed rank test was used. Spearman’s rank correlation was used to analyse correlations. Statistical significance was considered at *P*≤0.05.

## Results

### Impact of CHMI on vaccine-induced antibody responses

We have previously reported the induction of antigen-specific antibody responses following ChAd63-MVA immunization. In these Phase I/IIa clinical trials, healthy UK adults were immunized with the viruses encoding the MSP1, AMA1 or ME-TRAP antigens either alone or co-administered in various combinations [19]–[22] (Figure S1). In the Phase Ia safety and immunogenicity trials (Figure S1A), antibody responses against the MSP1_19_ and AMA1 antigens were shown to peak 4 weeks after the MVA boost (nominal trial day 84) [19], [20]. In the Phase IIa CHMI studies where volunteers were exposed to five infectious mosquito bites harbouring 3D7 clone *P. falciparum* parasites, volunteers were typically challenged 16 days after the MVA boost (Figure S1B). Volunteers, who did not exhibit sterile protection, typically developed microscopically patent blood-stage parasitemia within 9–14 days at which point they were drug-treated [21]. Here we report on the impact of blood-stage parasite exposure on vaccine-induced antibody responses and on the *de novo* induction of malaria-specific antibody responses in non-immunized infectivity control volunteers (Figure S1C).

**Figure 1:**
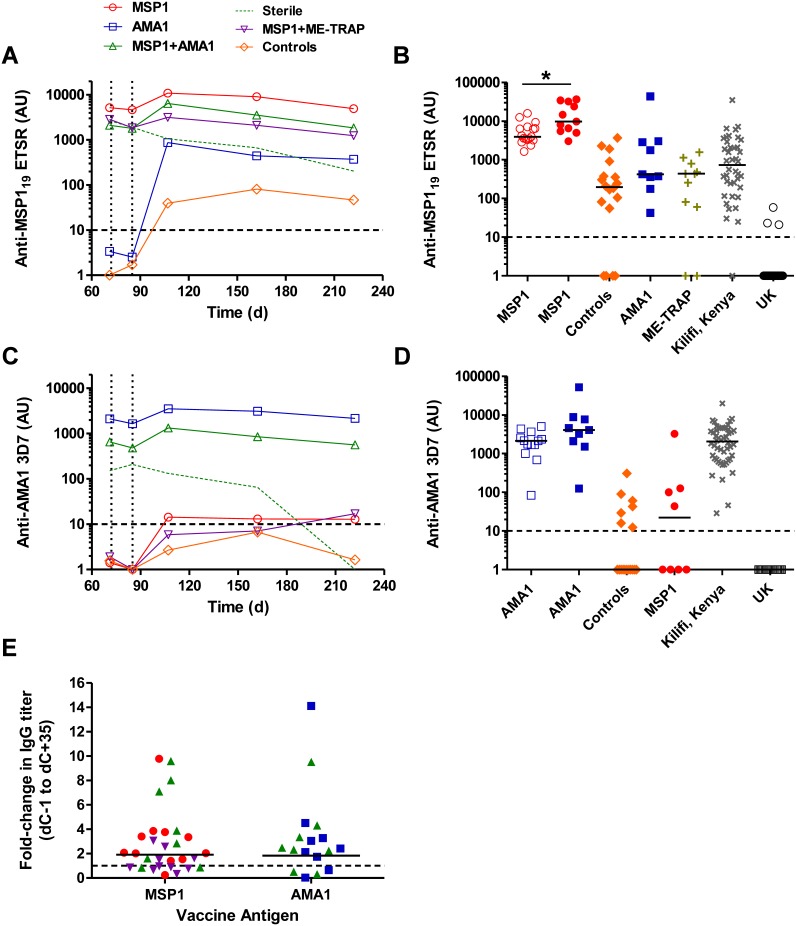
Assessment of IgG antibody responses post-CHMI. Mean anti-MSP1_19_ serum IgG responses were assessed over time by ELISA in a Phase IIa CHMI trial [21]. Dashed vertical lines represent: day 72 (d72) = day of CHMI; and d85 = nominal day of diagnosis. The first follow-up time-point after CHMI = day 107 (dC+35). (A) VAC039: MSP1 vaccinees (n = 8**–**9 depending on time-point assessed); AMA1-only vaccinees (n = 9); MSP1+AMA1 vaccinees (n = 8) plus one volunteer who was steriley protected in this group (dashed line); MSP1+ME-TRAP (n = 10); and infectivity controls (n = 6). The second Phase IIa CHMI trial with MSP1 vaccinees (VAC037) is shown in Figure S2. (B) Individual and median anti-MSP1_19_ serum IgG responses are shown for MSP1 vaccinees at the peak after the MVA boost (open symbols, n = 17) [19], [21] and at dC+35 following CHMI (closed symbols, n = 11) [21]; at dC+35 for 18 infectivity control volunteers from three separate CHMI studies [21], [22]; at dC+35 for AMA1 vaccinees (n = 9) [21] and non-sterilely protected ME-TRAP vaccinees (n = 11) [22]; from 40 naturally-exposed immune adults from Kilifi, Kenya; and 59 malaria-naïve UK adults (prior to immunization or CHMI). (C) Anti-AMA1 serum IgG responses for each group as in panel A. (D) Individual and median anti-AMA1 serum IgG responses are shown for AMA1 vaccinees at the peak after the MVA boost (open symbols, n = 13) [20], [21] and at dC+35 following CHMI (closed symbols, n = 9) [21]; at dC+35 for 18 infectivity control volunteers from three separate CHMI studies [21], [22]; at dC+35 for MSP1-only vaccinees (n = 8) [21]; from 50 naturally-exposed immune adults from Kilifi, Kenya; and 19 malaria-naïve UK adults (prior to immunization or CHMI). (E) The fold-change in IgG titer from dC−1 (d71) to dC+35 (d107) is reported. Individual responses and geomean are shown, with symbol colouring according to group in panels A and C. The limit of detection in both ELISA assays was 10 AU (dashed horizontal line), and we assigned the AU value of 1.0 for any test samples with less than 10 AU. Any values more than 10 AU are considered as positive responses. *P<0.05 (Wilcoxon matched-pairs signed rank test).

Antibody responses were monitored from the day before challenge (dC−1) in volunteers receiving ChAd63-MVA immunization encoding MSP1 alone, AMA1 alone, MSP1+AMA1 co-administered or MSP1+ME-TRAP co-administered (Figure 1A and Figure S2). dC−1 is plotted as nominal study day 71 (given CHMI occurred on average across all volunteers 16 days after the MVA booster immunization on day 56). dC−1 total IgG responses in vaccinees against MSP1_19_ and AMA1 have been previously reported [21] and are shown here for completeness. In two separate Phase IIa challenge studies (called VAC037 and VAC039 – Figure S2 and Figure 1A respectively), serum total IgG responses against MSP1_19_ tended to decline in MSP1 vaccinees up until the point of diagnosis (perhaps due to removal of antibody from circulation that binds to MSP1 antigen), but showed a significant increase at the first follow-up visit after drug treatment (dC+35/d107), *P* = 0.02 (Wilcoxon matched-pairs signed rank test, *n = *11 data pooled from both studies) (Figure 1A, B and Figure S2). Very similar results were observed in volunteers immunized with MSP1+AMA1 or MSP1+ME-TRAP (Figure 1A), although initial anti-MSP1_19_ antibody responses were lower given immune-interference following vaccine co-administration, as previously reported [21]. One out of nine volunteers immunized with MSP1+AMA1 showed sterile protection [21]. The results for this volunteer, who experienced no detectable blood-stage malaria infection, are plotted separately (Figure 1A, green dashed line). In agreement with the absence of blood-stage parasite exposure, there was no boosting following exposure and the anti-MSP1_19_ IgG response declined over time. Overall, across all infected MSP1 vaccinees (*n = *29), blood-stage parasite exposure led to a 1.9 fold increase (geometric mean, 95% CI: 1.3–2.7) in the anti-MSP1_19_ IgG antibody titer at dC+35 (Figure 1E).

In the case of the control volunteers (Figure S1C) [21], almost all became sero-positive against the MSP1_19_ antigen, with 5/6 in the first study (Figure S2) and 4/6 in the second study (Figure 1A) showing detectable IgG responses at dC+35. Pooling these data with more control volunteers from a third CHMI study [22], showed 14/18 volunteers with anti-MSP1_19_ responses at dC+35 (Figure 1B). Sero-positivity against MSP1_19_ tended to be slightly improved in volunteers previously immunized with AMA1 alone (Figure 1A, B), with 9/9 volunteers showing positive IgG responses at the same time-point. In contrast, volunteers receiving prior immunization with the pre-erythrocytic antigen ME-TRAP alone [22], showed a similar rate of sero-positivity to the controls, with 9/11 infected volunteers having measurable responses (Figure 1B).

Similar results were noted for AMA1 vaccinees. Volunteers previously immunized with this antigen and exposed to blood-stage parasites showed higher antibody responses at the dC+35 time-point (Figure 1C, D), and again, no boosting was observed in the one protected volunteer co-immunized with MSP1+AMA1 (Figure 1C). Across all infected AMA1 vaccinees (*n = *17), blood-stage parasite exposure led to a 1.8 fold increase (geometric mean, 95% CI: 0.9–3.8) in the anti-AMA1 IgG antibody titer at dC+35 (Figure 1E). In the case of the control volunteers and those immunized with MSP1 alone or MSP1+ME-TRAP, responses were on average marginal and just above the limit of detection of the assay (Figure 1C). In an analysis of 18 controls volunteers across three separate CHMI studies, only 7/18 showed sero-positivity at the dC+35 time-point, and only 4/8 following prior immunization with MSP1 (Figure 1D). The induction of *de novo* antibody responses against AMA1 thus appeared weaker in comparison to MSP1_19_.

Finally we compared these responses to those observed in sera taken from a cross-sectional bleed from naturally-exposed immune adults residing in Kilifi district, on the east coast of Kenya. Responses measured to both antigens following many years of natural exposure showed a broad spread in terms of magnitude, with the strongest responses similar to those induced by ChAd63-MVA immunization (Figure 1B, D).

### Associations between IgG antibody responses to MSP1_19_ and AMA1 and CHMI outcome measures

Having measured antibody responses post-CHMI in vaccinees and controls, we next sought to assess associations between the impact of CHMI on antibody responses and other outcome measures (Table 1). In the case of MSP1_19_, there was a strong significant correlation (*P* = 0.006) between the pre-challenge IgG antibody titer at dC−1 in volunteers receiving the MSP1 vaccine (either alone or co-administered) and the post-challenge IgG titer measured at dC+35. A similar trend was observed in AMA1 vaccinees, but did not reach significance (*P* = 0.06). However, there was no clear correlation between the dC−1 titer and subsequent fold-change in IgG titer (as shown in Figure 1E) for either antigen, nor was there any relationship between the fold-change in IgG titer and the time to blood-stage infection diagnosis by thick-film microscopy or the level of parasitemia, measured as parasites/mL blood by quantitative PCR (qPCR), at the time of diagnosis.

**Table 1:** Associations between IgG antibody responses and CHMI outcome measures.

Group	Comparison	MSP1_19_ ETSR IgG titer			AMA1 3D7 IgG titer		
		r_s_	*P*	n	r_s_	*P*	n
Vaccinees*	dC−1 vs dC+35	0.49	**0.006**	29	0.46	0.06	17
	dC−1 vs Fold-change post-CHMI	−0.08	0.68	29	−0.14	0.59	17
	Fold-change vs Time-to-Diagnosis	−0.02	0.91	29	−0.05	0.86	17
	Fold-change vs Parasitemia at Diagnosis	0.13	0.52	28	0.24	0.36	16
Controls	dC+35 vs Time-to-Diagnosis	0.59	**0.01**	18	0.36	0.14	18
	dC+35 vs Parasitemia at Diagnosis	0.52	**0.03**	18	0.40	0.10	18
AMA1-onlyVaccinated	dC+35 vs Time-to-Diagnosis	0.24	0.52	9		n/a	
	dC+35 vs Parasitemia at Diagnosis	0.58	0.11	9		n/a	
ME-TRAP-onlyVaccinated	dC+35 vs Time-to-Diagnosis	0.64	**0.03**	11		n.d.	
	dC+35 vs Parasitemia at Diagnosis	0.80	**0.003**	11		n.d.	
MSP1-onlyVaccinated	dC+35 vs Time-to-Diagnosis		n/a		0.26	0.54	8
	dC+35 vs Parasitemia at Diagnosis		n/a		0.51	0.20	8

Associations are reported between anti-MSP1_19_ or anti-AMA1 total IgG ELISA titer readouts at various time-points and/or fold-change post-CHMI (as in Figure 1E), as well as with time to malaria diagnosis by thick-film microscopy during CHMI, and parasitemia at time of diagnosis (measured by qPCR in terms of parasites/mL blood). In all cases, Spearman’s rank correlation coefficient and *P* value are shown. n/a = not applicable; n.d. = not done. *For these analyses, relevant vaccine groups were included: for the MSP1_19_ analysis, data were combined from the MSP1-only vaccination group and from the MSP1+AMA1 and MSP1+ME-TRAP co-administration groups; and for the AMA1 analysis data were combined from the AMA1-only vaccination group and from the MSP1+AMA1 co-administration group.

In the case of the unimmunized control volunteers and ME-TRAP vaccinees (both groups being ‘blood-stage antigen naïve’), there was a significant positive correlation between their dC+35 IgG titers against MSP1_19_ and time-to-diagnosis as well as parasitemia at diagnosis, thus suggesting the degree of parasite exposure strongly influences antibody induction against this antigen. In contrast, this was not observed in the AMA1 vaccinees, in agreement with the above observations that prior exposure to the blood-stage AMA1 antigen is affecting the *de novo* immunological response to MSP1_19_. As outlined above, *de novo* responses to AMA1 were much weaker in the controls and MSP1 vaccinated individuals, and similar relationships with CHMI outcome measures were not observed against this antigen.

We also assessed cross-reactivity by ELISA of these sera against other alleles of MSP1_19_ and AMA1. Both the MSP1 and AMA1 viral vectored vaccines expressed bi-allelic constructs. In the case of MSP1 both major alleles of MSP1_19_ were encoded [32], which differ by 4 amino acids – the ETSR allele (present in the 3D7 clone parasite used for CHMI studies) as well as the QKNG (or K1/Wellcome) allele. In the case of AMA1, two versions of the ectodomain were expressed [33] – the 3D7 and FVO alleles which differ by 24 amino acids. Following vaccination only (dC−1 sera in the Phase IIa trials), there was a highly significant correlation between ELISA responses against recombinant proteins for both alleles of both antigens (Table 2). This was in agreement with previous reported observations from the Phase Ia trials [19], [20] and similar trials of protein-based vaccines [40]. Following CHMI, and exposure to only 3D7 clone parasites, this highly significant correlation was maintained for both antigens in the vaccinated volunteers. In the case of the control volunteers, who only experienced 3D7 clone infection during the CHMI study, responses post-CHMI at dC+35 to the homologous ETSR allele of MSP1_19_ still showed a strong significant correlation with those measured against the heterologous QNKG allele, but the relationship was slightly weaker in comparison to the vaccinees who had been previously immunized against both. A similar result was observed in the immune sera from the Kenyan adults where a strong correlation in ELISA reactivity was observed against both alleles of MSP1_19_ and AMA1 in agreement with other studies [41].

**Table 2:** Associations between IgG antibody ELISA responses against two alleles of MSP1_19_ and AMA1.

Time-point	Group	MSP1_19_ ETSR vs QKNG			AMA1 3D7 vs FVO		
		r_s_	*P*	n	r_s_	*P*	n
dC−1	Vaccine*	0.93	**<0.0001**	18	0.97	**<0.0001**	18
dC+35	Vaccine + CHMI*	0.95	**<0.0001**	18	0.95	**<0.0001**	18
dC+35	CHMI only	0.79	**0.002**	12		n.d.	
n/a	Kilifi, Kenya	0.93	**<0.0001**	35	0.84	**<0.0001**	45

Associations are reported between anti-MSP1_19_ or anti-AMA1 total IgG ELISA titer readouts between two allelic variants of each antigen – ETSR (3D7) and QKNG (K1/Wellcome) for MSP1_19_, and 3D7 and FVO for AMA1. Responses were assessed following vaccination only, vaccination + CHMI, CHMI only (control volunteers) and in naturally-exposed Kenyan adults (as described in Figure 1B, D). In all cases, Spearman’s rank correlation coefficient and *P* value are shown. n.d. = not done (too few positive responders). *Relevant data were pooled for analysis from vaccinees receiving either a single vaccine or co-administered vaccines (as described in Table 1).

### Impact of CHMI on *in* *vitro* GIA

**Figure 2:**
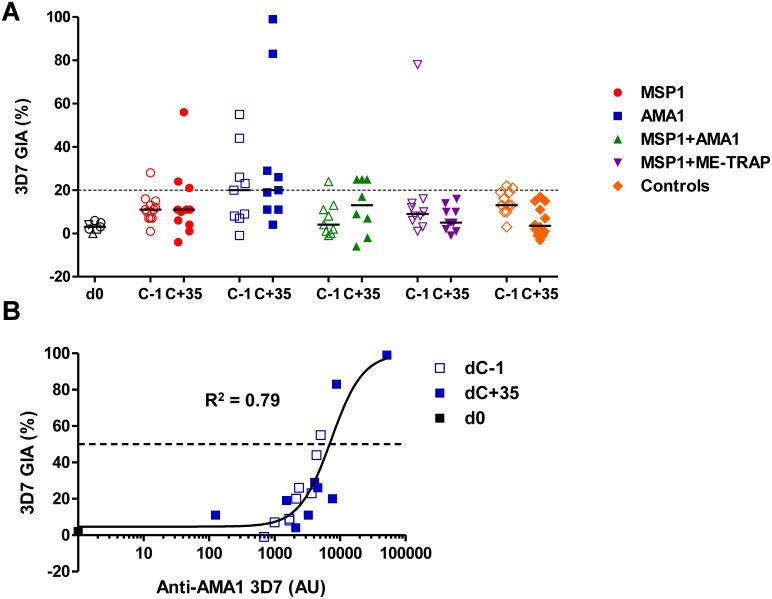
Assessment of functional GIA post-CHMI. (A) *In* ****** *vitro* GIA of purified IgG was assessed at 10****mg/mL against 3D7 clone *P. falciparum* parasites. Individual data and medians are shown for each vaccinated or control group at the dC−1 and dC+35 time-points (*n* = 8**–**12). Pre-immunization (d0) sera were either tested individually or pooled (*n* = 7). Responses >20% are typically regarded as positive. (B) Relationship between GIA and anti-AMA1 serum IgG responses measured by ELISA. Results for volunteers immunized with ChAd63-MVA AMA1 are shown on dC−1 (before CHMI) and dC+35 (after CHMI). Non-linear regression curve is also shown (*n* = 19). The level of AMA1 antibody measured in this ELISA assay that gave 50% GIA (EC_50_, dashed black line) was 7294 AU (95% C.I. = 3981**–**13362).

Having shown the impact of CHMI on vaccine-induced and *de novo* IgG antibody responses, we also sought to assess the impact on functional activity. *In vitro* GIA was measured against challenge homologous 3D7 clone parasites using 10 mg/mL purified IgG from the dC−1 and dC+35 time-points. The dC−1 data have been previously reported [21] and are included here for ease of comparison. Following CHMI, there was no change in the average levels of GIA between dC−1 and dC+35 (the peak of the serum antibody response) in any group (Figure 2A). No measurable GIA was induced in the controls following CHMI. A small handful of volunteers showed increased responses, in particular purified IgG from two AMA1 vaccinees showed 83% and 99% GIA at dC+35. The levels of GIA in the purified IgG from all the AMA1 vaccinees also showed a strong relationship with anti-AMA1 IgG antibody titer (Figure 2B).

### Analysis of anti-MSP1_19_ and -AMA1 antibody avidity following vaccination, CHMI and natural exposure

**Figure 3:**
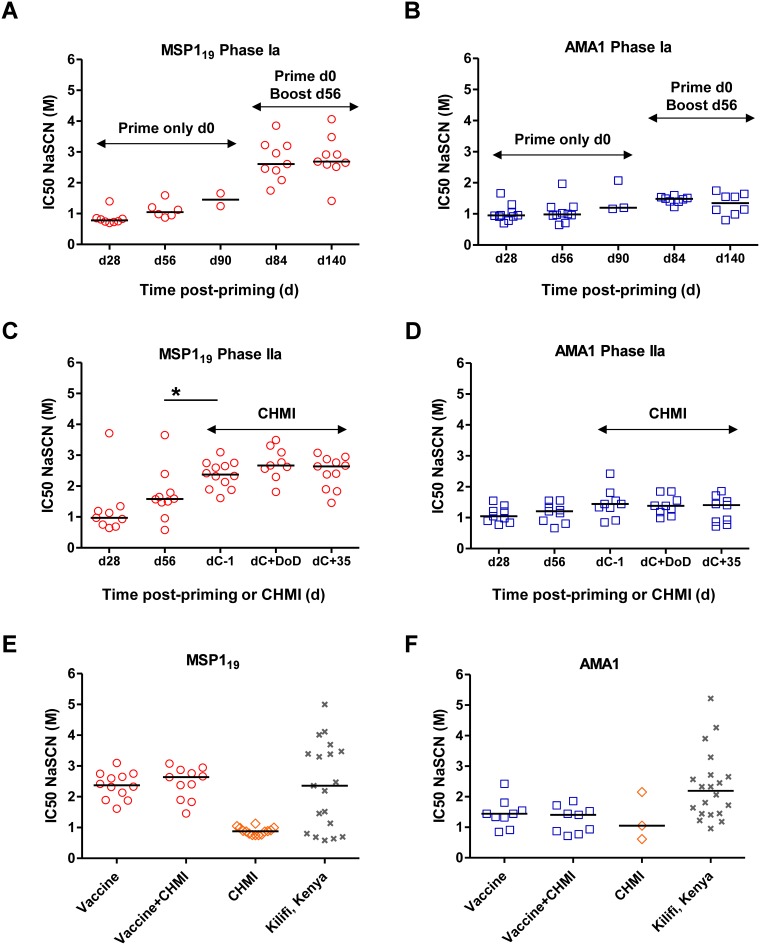
Assessment of IgG avidity following vaccination, CHMI and natural exposure. Avidity of serum IgG responses was assessed by NaSCN-displacement ELISA and is reported as the molar concentration of NaSCN required to reduce the OD405 to 50% of that without NaSCN (IC50). Where samples at specific time-points tested negative for antigen-specific total IgG responses by ELISA or these responses were too low to analyse, the avidity is not reported. Individual responses are shown, plus the median of the positive samples (i.e. those with a detectable antigen-specific response in the total IgG ELISA that enabled assessment for avidity). (A) Anti-MSP1_19_ responses in the VAC037 Phase Ia clinical trial [19] following ChAd63 MSP1 priming immunization (at d28, d56 and d90) or following the MVA MSP1 boost (d84 and d140). (B) Anti-AMA1 responses in the VAC036 Phase Ia clinical trial [20], reported as in panel A. (C) Anti-MSP1_19_ responses in the VAC039 Phase IIa clinical trial [21] following ChAd63 MSP1 priming immunization (at d28 and d56); or following the MVA MSP1 boost (dC−1, equivalent to d84 in panel A); or following CHMI at day of diagnosis, dC+DoD, or at first follow-up post-drug treatment, dC+35. (D) Anti-AMA1 responses in the VAC039 Phase IIa clinical trial [21], reported as in panel C. In panels A–D, *n* = 2**–**12 depending on sample availability for each tested time-point. (E) Individual and median anti-MSP1_19_ serum IgG avidity responses are shown for MSP1-only vaccinees at the peak after the MVA MSP1 boost (“Vaccine”, *n* = 12) and at dC+35 following CHMI (“Vaccine+CHMI”, *n* = 11) [21]; at dC+35 for 14 infectivity control volunteers from three separate CHMI studies (“CHMI”) [21], [22]; and from 19 naturally-exposed immune adults from Kilifi, Kenya. (F) Individual and median anti-AMA1 serum IgG avidity responses are shown for AMA1-only vaccinees at the peak after the MVA AMA1 boost (*n* = 9) and at dC+35 following CHMI (*n* = 9) [21]; and for 3 infectivity control volunteers and 20 naturally-exposed immune adults from Kilifi, Kenya. *P<0.05 (Wilcoxon matched-pairs signed rank test).

We next assessed the avidity of the anti-MSP1_19_ and -AMA1 antibodies using a NaSCN-displacement ELISA. Initially this was measured in serum from volunteers in the Phase Ia clinical trials of these vaccines [19], [20]. Responses were assessed at d28, d56 and d90 for those receiving ChAd63 MSP1 or AMA1 vaccines alone, and at d28, d56, d84 and d140 for those that were also boosted with MVA encoding the same antigen at d56 (Figure S1A). For MSP1_19_ the median avidity of the antibodies tended to increase slightly over time following ChAd63 MSP1 priming (from d28 to d90) (Figure 3A). Administration of the MVA MSP1 boost at day 56 led to an increase in IgG avidity, with no further increase evident over time as assessed out to d140. In contrast for AMA1, there was relatively little, if any, change over time following the prime (Figure 3B). After the MVA AMA1 boost at day 56 there was an increase in median avidity, however, the change in NaSCN IC50 was extremely small in comparison to that observed with MSP1_19_.

In the Phase IIa challenge study, we similarly measured IgG avidity after vaccination and also after blood-stage parasite exposure [21]. In these new groups of volunteers, we observed similar changes in avidity measured after the MVA boost vaccinations, as to those seen previously in the Phase Ia trials. When comparing day 56 with dC−1 (equivalent to day 84 in the Phase Ia studies), there was a significant increase in avidity for MSP1_19_ (*P* = 0.03, Wilcoxon matched-pairs signed rank test, *n = *9) (Figure 3C) and another very small increase in the median IC50 for AMA1 (Figure 3D). Following CHMI, IgG avidity was measured on the day of malaria diagnosis (dC+DoD) and 35 days post challenge (dC+35). There was no change in the avidity of the antibodies when comparing the post-challenge time-points with dC−1, despite parasite exposure and boosting of the IgG titers (Figure 1).

We next assessed the avidity of antibodies against MSP1_19_ and AMA1 in 20 adults from the cohort in Kilifi, Kenya as well as in the infectivity control volunteers from the CHMI studies. The antigen-specific IgG in the Kenyan adults showed a much greater range of avidity in comparison to the vaccinees, ranging from 0–5 M for both MSP1_19_ (Figure 3E) and AMA1 (Figure 3F); indicating that much higher avidities can be achieved following natural exposure (potentially a reflection of life-long multiple exposures) as opposed to vaccination and a single infectious exposure in the context of CHMI. Few volunteers had anti-AMA1 IgG responses post-CHMI, but the few that did showed similar avidities to the vaccinees (Figure 3F). In contrast, most of the CHMI controls became sero-positive to MSP1_19_, and these serum IgG responses showed relatively low avidity in comparison to vaccination (Figure 3E).

Finally we assessed for any relationships between antigen-specific IgG avidity and total IgG titer as measured by these ELISA assays. Across all the groups studied – irrespective of vaccination status, or parasite exposure via CHMI or natural infection, we observed no significant correlations between IgG avidity and titer (Table 3), suggesting these assays measure independent attributes of the antibody response.

**Table 3:** Associations between IgG antibody avidity and total IgG ELISA titer.

Time-point	Group	MSP1_19_ ETSR			AMA1 3D7		
		r_s_	*P*	n	r_s_	*P*	n
dC−1	Vaccine*	−0.50	0.10	12	0.57	0.12	9
dC+35	Vaccine + CHMI*	−0.17	0.61	11	−0.02	0.98	9
dC+35	CHMI only	0.37	0.20	14		n.d.	
n/a	Kilifi, Kenya	0.01	0.97	19	0.28	0.23	20

Associations are reported between anti-MSP1_19_ ETSR allele or anti-AMA1 3D7 allele total IgG ELISA titer readouts (Figure 1B, D) and the corresponding IgG avidity measurement by NaSCN-displacement ELISA (Figure 3E, F). Groups were assessed according to vaccination and/or parasite exposure status as outlined in Figure 3E, F. In all cases, Spearman’s rank correlation coefficient and *P* value are shown. n.d. = not done (too few positive responders). *Data from vaccinees receiving only a single vaccine (i.e. MSP1-only or AMA1-only) were used for this analysis.

### Analysis of anti-MSP1_19_ and -AMA1 antibody isotypes following vaccination, CHMI and natural exposure

**Figure 4:**
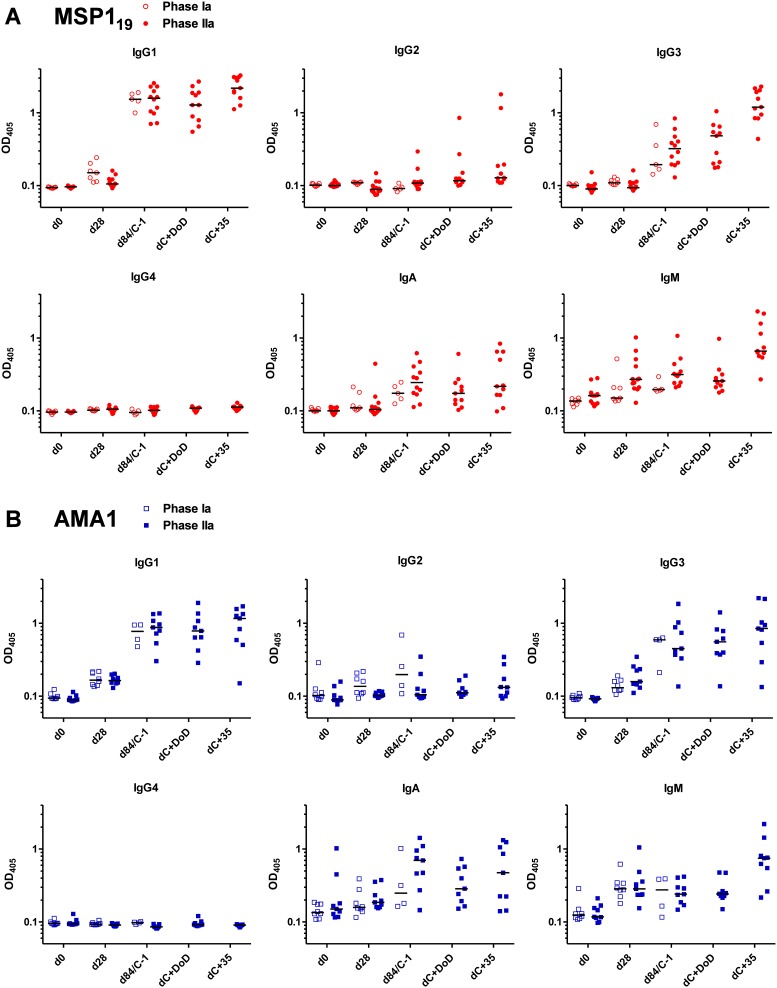
Assessment of antibody isotype profiles following vaccination and CHMI. Isotype profiles of serum antibody responses were assessed by ELISA. (A) Anti-MSP1_19_ responses in the VAC037 Phase Ia clinical trial (open symbols) [19] and the VAC039 Phase IIa clinical trial (closed symbols) [21]. Responses are shown at baseline (d0); following ChAd63 MSP1 priming immunization (d28); following the MVA MSP1 boost (d84 in the Phase Ia trial or dC−1 in the Phase IIa trial); or following CHMI at day of diagnosis, dC+DoD, or at first follow-up post drug treatment, dC+35. (B) Anti-AMA1 responses in the VAC036 Phase Ia clinical trial [20] and VAC039 Phase IIa trial [21], reported as in panel A. In all panels, individual and median responses are shown, n = 4**–**13 depending on sample availability for each tested time-point.

Similar to the IgG avidity analysis, we also examined the serum antibody isotype profile to both antigens by ELISA. Initially this was measured in serum from volunteers immunized with ChAd63-MVA MSP1 or AMA1 in the Phase I/IIa clinical trials of these vaccines [19]–[21]. Results were comparable across the cohorts of volunteers immunized with the same vaccines in the different clinical trials (open versus closed symbols, Figure 4A, B). Following the MVA boost, IgG1 and IgG3 were the predominant IgG isotypes against both MSP1_19_ and AMA1. IgG2 was detectable in a minority of volunteers, and no IgG4 response was detected against either antigen. IgA and IgM responses were also detected to both antigens in the majority of volunteers (Figure 4A, B). Exposure to 3D7 clone blood-stage parasites in the context of CHMI showed minimal impact on the isotype profile during infection (assessed at the diagnosis time-point) or following treatment (dC+35). There were notable increases in the levels of IgG1, IgG3 and IgM in both the MSP1 and AMA1 vaccinees by dC+35 (Figure 4A, B).

**Figure 5:**
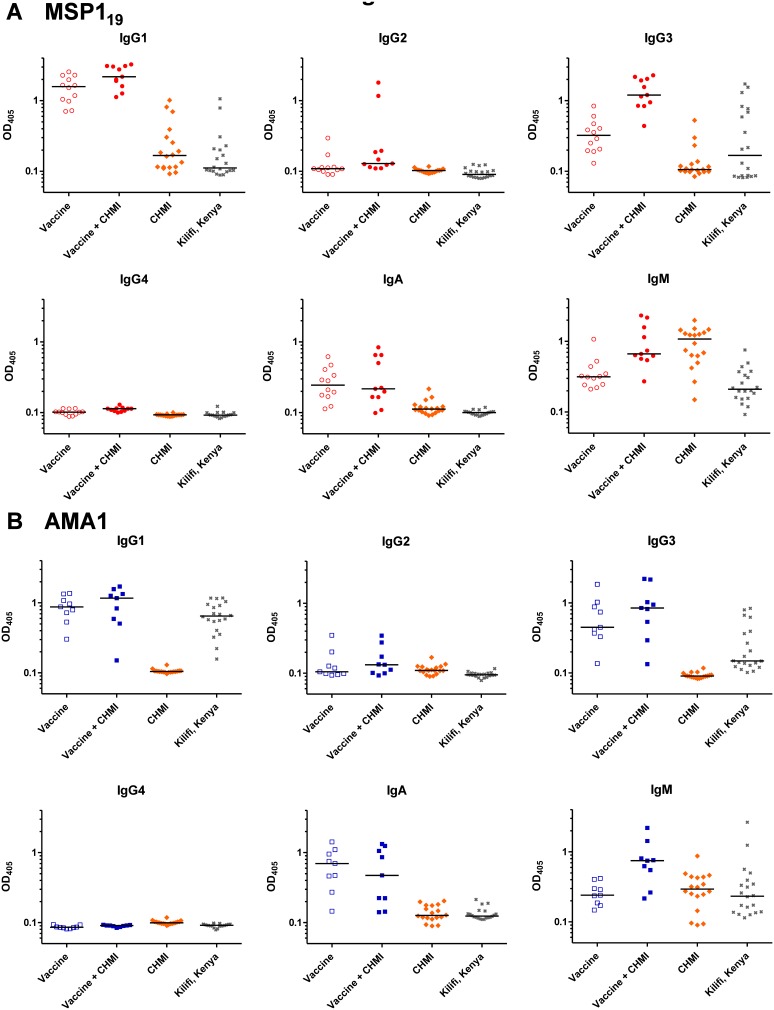
Assessment of antibody isotype profiles following vaccination, CHMI and natural exposure. Isotype profiles of serum antibody responses were assessed by ELISA. (A) Individual and median anti-MSP119 serum antibody isotype responses are shown for MSP1-only vaccinees at the peak after the MVA MSP1 boost (“Vaccine”, *n* = 12) and at dC+35 following CHMI (“Vaccine+CHMI”, *n* = 11) [21]; at dC+35 for 18 infectivity control volunteers from three separate CHMI studies [21], [22]; and from 20 naturally-exposed immune adults from Kilifi, Kenya. (B) Individual and median anti-AMA1 serum antibody isotype responses are shown for AMA1-only vaccinees at the peak after the MVA AMA1 boost (*n* = 9) and at dC+35 following CHMI (*n* = 9) [21]; and for infectivity control volunteers and naturally-exposed immune adults from Kilifi, Kenya as in panel A.

We also assessed the isotype profile of anti-MSP1_19_ and -AMA1 antibodies in the infectivity control volunteers from the UK who were challenged across three different mosquito bite CHMI studies. Similar to the vaccinees, following the low-density blood-stage infection, control volunteers who became sero-positive to MSP1_19_ showed predominantly IgG1 responses with some IgG3 (Figure 5A). There was no detectable IgG2 or IgG4, and only a few volunteers with low-level IgA. Interestingly, almost all volunteers showed an IgM response, stronger than that seen in the MSP1 vaccinees and in line with a primary humoral response to this antigen. A similar analysis of the naturally-immune adults from Kilifi, Kenya showed largely similar results, with a pre-dominance of IgG1 and IgG3; no detectable IgG2, IgG4 or IgA; and low-level IgM (Figure 5A).

Results with AMA1 were consistent with those observed for MSP1_19_. As noted earlier in the total IgG ELISAs (Figure 1D), few control volunteers showed detectable responses. In agreement with the less-sensitive isotype ELISAs, no IgG isotype responses were detected following CHMI alone, however, the majority of volunteers possessed serum anti-AMA1 IgM consistent with the primary response to infection (Figure 5B). The same analysis of the naturally-immune adults from Kilifi, Kenya again showed a pre-dominance of IgG1 with lower levels of IgG3 and IgA; no detectable IgG2 or IgG4; and low-level IgM (Figure 5B).

**Figure 6:**
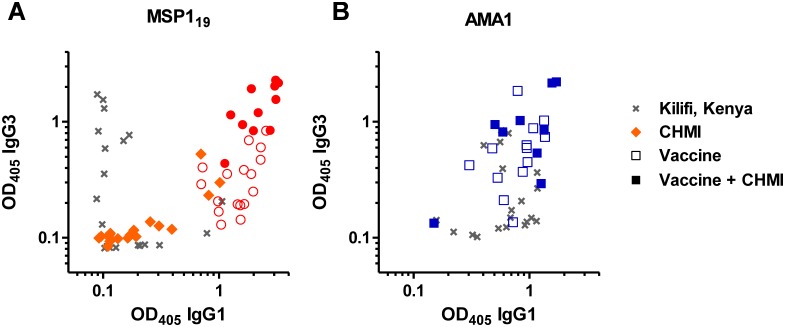
Correlations between anti-MSP119 and –AMA1 IgG1 and IgG3 isotype ELISA responses in different exposure populations. Serum IgG1 and IgG3 isotype responses were assessed by ELISA against (A) MSP119 (ETSR allele) and (B) AMA1 (3D7 allele). Correlations are shown: following MSP1 (n = 17) or AMA1 (*n* = 13) viral vectored vaccination only d84/dC−1 sera (open circle and square symbols respectively); following MSP1 (*n* = 11) or AMA1 (*n* = 9) vaccination followed by CHMI dC+35 sera (closed circle and square symbols respectively); following CHMI only (*n* = 18 for MSP119 only) in control volunteers’ dC+35 sera (closed diamond symbols); and for immune sera from Kenyan adults (*n* = 20; cross symbols).

Finally we assessed the relationship between the dominant IgG1 and IgG3 isotype responses against both antigens in the different populations. In the case of MSP1_19_, following vaccination alone (dC−1) and vaccination plus 3D7 clone parasite exposure in the context of CHMI (dC+35), immune sera showed a significant positive correlation between IgG1 and IgG3 antibodies (*P* = 0.04, r_s_ = 0.50, *n = *17; *P* = 0.02, r_s_ = 0.70, *n = *11, respectively) (Figure 6A). A similar observation was seen following CHMI only in the infectivity controls (*P* = 0.001, r_s_ = 0.79, *n = *18). Interestingly, a very contrasting relationship was observed in the Kenyan adults, where a very dichotomous response was observed – immune sera showed strong IgG1 or IgG3 responses, but not both (Figure 6A).

In the case of AMA1, as for MSP1_19_, we observed a significant correlation between IgG1 and IgG3 responses following vaccination (*P* = 0.03, r_s_ = 0.60, *n = *13). Following CHMI, this relationship was not as strong and did not reach significance (*P* = 0.11, r_s_ = 0.58, *n = *9) Similar to MSP1_19_, a different result was also observed in the Kenyan adults with all showing detectable anti-AMA1 IgG1 responses, but only about half of these also positive for anti-AMA1 IgG3. Overall these results suggest a propensity of the ChAd63-MVA viral vectored vaccines to induce a balanced IgG1 and IgG3 response, in comparison to parasite exposure where more skewed IgG isotype profiles appear evident.

## Discussion

This study has explored the impact of CHMI on qualitative and quantitative vaccine-induced antibody responses against two blood-stage malaria antigens – MSP1_19_ and AMA1. Initial data showed that a single CHMI (whereby all but one volunteer developed blood-stage infection) led to increased serum IgG antibody responses against both antigens in vaccinees post-infection and drug-treatment. On average, serum IgG responses to both antigens showed a similar 2-fold increase by 35 days post-CHMI. This kinetic and short-term re-boosting is consistent with modest incremental increases in antibody levels with age that are observed in repeatedly exposed individuals [42], [43], and also consistent with boosted memory B cell responses previously reported in these Phase IIa studies [27]. The significant relationship between pre- and post-challenge serum IgG titers for both antigens agrees with the fairly similar fold-change in antibody titers observed across all vaccinees, and suggests a fairly consistent impact of low-density blood-stage parasite exposure on B cell re-stimulation. Other Phase IIa CHMI studies of blood-stage vaccine candidates (protein-in-adjuvant or virosomes) have reported post-challenge antibody responses that are not boosted [44]–[46] or decline following CHMI [11]. In contrast, another Phase IIa study of a poxvirus-based vector (NYVAC) encoding seven *P. falciparum* malaria antigens reported an increase in sero-reactivity to MSP1 and AMA1 in vaccinees post-challenge [47], similar to that observed here in ChAd63-MVA vaccinees. In contrast to protein vaccines, these results may suggest that viral vector vaccination primes an antibody response that is better suited to boosting by natural malaria exposure, perhaps due the improved elicitation of cellular immune responses by viral vectors [17], [20], [28] that may provide improved CD4^+^ T cell help to B cells responding to the infection. We have previously characterized in detail the T cell responses induced by these vaccines following vaccination and CHMI [28], and on-going work is seeking to assess peripheral CD4^+^ T cells with a T follicular helper (Tfh) cell-like phenotype [48].

As previously observed in our study of B cell responses [27], following CHMI of blood-stage malaria-naïve control volunteers, almost all became sero-positive in terms of anti-MSP1_19_ IgG. Similar results have been reported in travellers returning with *P. falciparum* infection [49]. Development of these responses was strongly associated with the level of parasitemia at diagnosis, suggesting a relationship with the amount of MSP1 antigen exposure and/or the more abundant nature of this surface protein in comparison to the micronemal AMA1 antigen. MSP1_19_ sero-positivity in AMA1 vaccinees was also more pronounced, suggesting that pre-existing immune responses against a blood-stage antigen (in this case either anti-AMA1 antibodies or AMA1-specific CD4^+^ T cell help) may aid the induction of *de novo* responses against other blood-stage antigens. A previous report from a different series of CHMI studies suggested PfEMP1 and the R2 region of glutamate-rich protein (GLURP) may be similarly immuno-dominant to MSP1_19_, whilst responses against the R0 and R1 regions of GLURP, MSP3 C-terminus, F2 region of EBA175, and RIFINs are more akin to those observed against AMA1 [50].

Despite the induction of these *de novo* responses in malaria-naïve controls, and boosting of vaccine-induced responses in the MSP1 and AMA1 vaccinees, there was no significant impact on overall functional GIA activity of purified IgG assessed at the peak of the responses 35 days post-CHMI. Negative GIA in the serum of control volunteers after CHMI is consistent with a previous Phase IIa study report [44], although the study in returning travellers suggested some functional activity due to MSP1_19_ responses measured using an alternative assay with a chimeric parasite [49]. In the case of the AMA1 vaccinated group, GIA was associated with anti-AMA1 ELISA titer both pre- and post-CHMI consistent with the notion that the measurable activity was due to these antibodies as opposed to *de novo* responses against other blood-stage parasite antigens. A previous AMA1 vaccine study in Mali suggested that malaria-specific non-AMA1 IgGs can interfere with GIA mediated by AMA1-specific IgGs [51]. In our case however, there was no obvious induction of *de novo* antibodies against other antigens during the CHMI that interfered with AMA1-associated GIA. Nevertheless, given the overall low levels of GIA induced by ChAd63-MVA MSP1 or AMA1 immunization (a reflection of moderate IgG concentrations that are on average about 40 µg/mL antigen-specific IgG in humans – as reported and discussed elsewhere [19], [20], [39]), our on-going clinical work has assessed the merits of combining viral vectored and protein-in-adjuvant vaccines [17], [34], [52], [53] against AMA1 aiming to achieve even higher concentrations of antigen-specific IgG. These results and a quantitative analysis of antigen-specific IgG concentrations are reported elsewhere [54].

The role of IgG avidity in protection against the blood-stage merozoite remains a poorly understood attribute of antibody function. The NaSCN-displacement ELISA employed here represents a relatively crude measure of the overall avidity (sum of affinities) of the polyclonal antigen-specific IgG response. Interestingly, increased avidity was clearly evident following the MVA booster vaccination for anti-MSP1_19_ IgG responses, whereas changes were minimal at best following the boost with MVA-AMA1. These data would suggest substantial affinity maturation can occur following MVA booster vaccine administration, but this may well be antigen-dependent. A previous single allele AMA1 protein vaccine trial in healthy Dutch adults using three different adjuvants (Alhydrogel, Montanide ISA 720 and AS02) showed that neither adjuvant nor vaccine dose significantly influenced average avidity measured in the same manner as here [55]. Indeed the reported avidities (ranging on average from 0.6–0.9 M [55]) were only slightly lower than those observed here. In our study, subsequent malaria exposure in vaccinated individuals had no apparent effect on avidity for either antigen, although interestingly malaria exposure of control volunteers gave rise to a lower avidity anti-MSP1_19_ response (in comparison to that induced by vaccination), suggesting the IgG response underwent minimal affinity maturation in this context of primary low-density infection. In contrast, a wide range of avidities was observed for both antigens in the sera from Kenyan adults, similar to observations in rodent malaria models [56]. These data also confirmed that higher avidity responses can be achieved by a lifetime of natural exposure, although responses of these magnitudes were not achieved either by vaccination and/or a single CHMI. The extent to which such a parameter contributes to natural anti-merozoite immunity, however, is not understood. Responses against AMA1 and MSP1_19_ measured in this Phase IIa vaccine trial did not, by themselves, impact on parasite multiplication rates following CHMI with homologous 3D7 clone parasites delivered by mosquito bite [21]. Given ‘avidity’ is a term used to encompass the net contribution of antibody affinity versus valency versus epitope density to antibody-antigen binding, future studies may be more informative by attempting to measure these independent contributions at the clonal level [57]. Indeed, with regard to individual antibody affinity, the rate at which merozoite invasion is neutralized is most likely determined by the antibody on-rate and concentration, rather than off-rate [58]. Although an association between polyclonal antibody off-rate and risk of disease was recently reported in an immuno-epidemiological study of natural immunity to malaria [59], other studies of monoclonal antibodies against merozoite antigens have suggested rapid binding of antibody to merozoite antigens is associated with improved neutralization of RBC invasion *in vitro* [60].

The analysis of serum antibody isotypes showed that ChAd63-MVA MSP1 or AMA1 immunization in healthy UK adults induced predominantly IgG1, IgG3, IgA and IgM against both antigens. No role has been described for IgA in protection against blood-stage merozoites [38], however the induction of this isotype may be more relevant to the clinical development of the adenovirus-MVA vaccine platform against mucosal pathogens including HIV-1 [15], [17]. The strong induction of the cytophilic human IgG1 and IgG3 isotypes is consistent with a viral-based vaccine delivery platform [61], [62], and would also be of relevance to other pathogen targets, including HIV-1, where antibody-mediated cellular effector functions or specific IgG isotypes may be associated with protection [63]. In the context of blood-stage malaria immunity, the interaction of IgG1 and IgG3 with monocytes has been reported to be important for mediating effective antibody-dependent cellular inhibition (ADCI) via monocytes when using antibodies present in the serum of naturally exposed individuals [64]–[66] or those specific for antigens such as MSP3 or GLURP [67]–[69]. Indeed, IgG2 and IgG4 responses have also been shown to interfere with the opsonizing effects of IgG1 and IgG3 [65], [70]. However, the extent to which these activities could contribute to effective vaccine-induced immunity *in vivo* in humans remains widely debated. Complicated interplay has also been described between different antibody isotype Fc regions, activatory and inhibitory Fc receptors on immune cells, the complement system, and arrayed antigen within soluble immune complexes [71], [72], suggesting that elucidating specific roles of various antibody isotypes in effective immunity may be particularly difficult.

With regard to vaccine-induced AMA1- and MSP1-specific antibody isotype responses, a bivalent AMA1 protein vaccine administered in Alhydrogel elicited predominantly IgG1 [40], [73], with very similar results for a MSP1_42_ vaccine given in the same manner [74]. In the aforementioned vaccine trial (in healthy Dutch adults administered AMA1 protein formulated in three different adjuvants), these platforms induced high levels of IgG1, lower but similar levels of IgG3 and IgG4, and even lower but detectable levels of IgG2 [55]. The induction by protein-in-adjuvant vaccines of IgG2 and IgG4 may represent a fundamental difference between these adjuvants and viral vectors, as noted previously in comparative mouse studies [52]. Establishing vaccine platforms that can induce specific antibody isotype profiles in humans will likely remain relevant to on-going vaccine development efforts against a variety of diseases. In the context of natural malaria immunity, IgG1 and IgG3 responses against merozoite antigens or infected RBC, including IgG1 against MSP1 and IgG3 against AMA1 [75], [76], have been most often associated with clinical protection in immuno-epidemiological studies [75], [77], [78], with one report also associating IgG2 with protection [79]. Here we observed a mixed IgG1 and IgG3 response following vaccination with MSP1 and AMA1, which was maintained post-CHMI. A single CHMI induced predominantly IgG1 to MSP1_19_, and substantial levels of IgM to both antigens in agreement with a primary immune response. The Kenyan adults showed mixed IgG1 and IgG3 responses against AMA1 that were more comparable to vaccination, in contrast to MSP1_19_ where a dichotomous relationship was observed. Data are conflicting from other endemic populations, but in general IgG1 and/or IgG3 are most consistently observed for anti-AMA1 and anti-MSP1_19_ serum antibody responses [41], [43], [76], [80]. Polarization towards one isotype has been reported with increasing age (and malaria exposure) in some studies for merozoite antigens [81], but not others [76]. The tendency to produce IgG1 or IgG3 appears likely to reflect a complex interplay of immune system maturity, duration of antigen exposure and inherent characteristics of the antigen sequence [81].

Overall these data describe complex differences in qualitative aspects of human antibody responses against malaria antigens depending on the means of their induction and/or exposure of the host to the malaria parasite. Given the continued clinical development of viral vectored vaccines for a wide variety of diseases [15], [17] and other malaria antigens [23], [82], [83], these data should help to guide further immuno-monitoring studies of vaccine-induced human antibody responses.

## Supporting Information

Figure S1 Trial timelines. (A) In Phase Ia studies, the ChAd63 priming vaccination was administered on day 0 (d0) and the MVA booster vaccination on day 56 (d56). Blood samples were taken for exploratory immunology at the indicated time-points. (B) In Phase IIa studies, volunteers were immunized in the same manner. Subsequently, controlled human malaria infection (CHMI) followed the MVA booster vaccination on average 16 days later (range 13–25 days). Blood samples were taken at the indicated time-points, including the day before CHMI (dC−1); 35, 90 and 150 days post-CHMI (dC+35, etc.); and at the time-point of malaria diagnosis (dC+DoD), nominally d85. (C) Phase IIa infectivity control volunteers underwent CHMI without previous vaccination in parallel with vaccinated volunteers. Non-protected and control volunteers were diagnosed with microscope-patent blood-stage malaria infection on average 10–11 days after CHMI (dC+10/11) at which point they received anti-malarial drug treatment [21]. The next follow-up time-point was dC+35.
(TIF)

Figure S2 Assessment of MSP119 IgG antibody responses post-CHMI. Mean anti-MSP119 serum IgG responses were assessed over time by ELISA and are shown for a second Phase IIa CHMI trial [21]. Dashed vertical lines represent: day 72 (d72) = day of CHMI; and d85 = nominal day of diagnosis. The first follow-up time-point after CHMI = day 107 (dC+35). The data are shown for the VAC037 trial: MSP1 vaccinees (n = 3); and infectivity controls (n = 6). The limit of detection in the ELISA assay was 10 AU (dashed horizontal line), and we assigned the AU value of 1.0 for any test samples with less than 10 AU. Any values more than 10 AU are considered as positive responses.
(TIF)
