# Supplementary figures and images for: Assessment of Humoral Immune Responses to Blood-Stage Malaria Antigens following ChAd63-MVA Immunization, Controlled Human Malaria Infection and Natural Exposure
Source: PLoS One. 2014 Sep 25;9(9):e107903. doi: 10.1371/journal.pone.0107903 (PMC4177865; doi:10.1371/journal.pone.0107903)

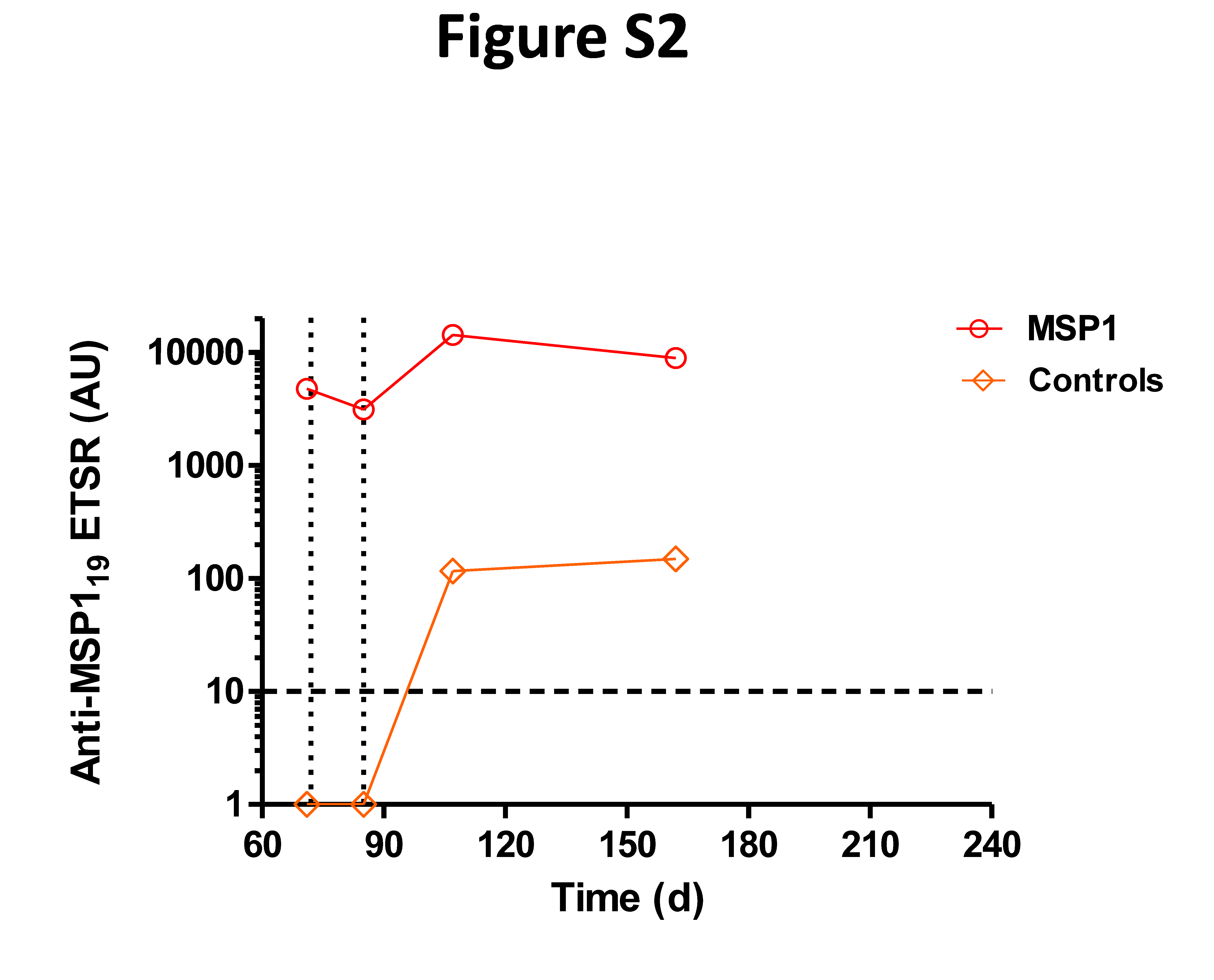

Supplement: Figure S2 — Assessment of MSP119 IgG antibody responses post-CHMI. Mean anti-MSP119 serum IgG responses were assessed over time by ELISA and are shown for a second Phase IIa CHMI trial [21]. Dashed vertical lines represent: day 72 (d72) = day of CHMI; and d85 = nominal day of diagnosis. The first follow-up time-point after CHMI = day 107 (dC+35). The data are shown for the VAC037 trial: MSP1 vaccinees (n = 3); and infectivity controls (n = 6). The limit of detection in the ELISA assay was 10 AU (dashed horizontal line), and we assigned the AU value of 1.0 for any test samples with less than 10 AU. Any values more than 10 AU are considered as positive responses. (TIF) [file pone.0107903.s002.tif]
